# A Novel Surface Electromyographic Signal-Based Hand Gesture Prediction Using a Recurrent Neural Network

**DOI:** 10.3390/s20143994

**Published:** 2020-07-17

**Authors:** Zhen Zhang, Changxin He, Kuo Yang

**Affiliations:** School of Mechatronic Engineering and Automation, Shanghai University, Shanghai 200444, China; hcx1995@shu.edu.cn (C.H.); yangkuohao@shu.edu.cn (K.Y.)

**Keywords:** sEMG, hand gesture prediction, RNN, myo armband

## Abstract

Surface electromyographic signal (sEMG) is a kind of bioelectrical signal, which records the data of muscle activity intensity. Most sEMG-based hand gesture recognition, which uses machine learning as the classifier, depends on feature extraction of sEMG data. Recently, a deep leaning-based approach such as recurrent neural network (RNN) has provided a choice to automatically learn features from raw data. This paper presents a novel hand gesture prediction method by using an RNN model to learn from raw sEMG data and predict gestures. The sEMG signals of 21 short-term hand gestures of 13 subjects were recorded with a Myo armband, which is a non-intrusive, low cost, commercial portable device. At the start of the gesture, the trained model outputs an instantaneous prediction for the sEMG data. Experimental results showed that the more time steps of data that were known, the higher instantaneous prediction accuracy the proposed model gave. The predicted accuracy reached about 89.6% when the data of 40-time steps (200 ms) were used to predict hand gesture. This means that the gesture could be predicted with a delay of 200 ms after the hand starts to perform the gesture, instead of waiting for the end of the gesture.

## 1. Introduction

Hand gesture recognition is a promising human-computer interaction, which is widely discussed and studied in various areas. The capability of machines to recognize distinctive gesture characteristics can be harnessed in a wide variety of applications including the control of bionic hands [1,2], virtual game control [3], sign language translation [4], smart wheelchair [5], and intelligent robotics [6,7]. To this day, various sensors have been used to recognize hand gesture. Data glove that contains bending sensors and accelerometers are able to capture the rotation and movement of the hand and fingers [8], while it is not convenient and unnatural to wear a cumbersome glove in daily life. Cameras are also used to recognize hand motion [9], while it is sensitive to the use environment such as background texture, color, and lighting. Surface electromyographic signal (sEMG) is a useful non-intrusive technique for recording the electrical activity produced by muscles through surface sensors placed on the skin, which is a promising candidate for motion detection, gesture recognition and even gesture prediction [10,11,12]. 

In previous studies, a variety of features of sEMG signal were designed and extracted to classify hand gesture [13,14,15,16,17,18,19,20]. These features included mean absolute value, zero crossing, root mean square, power spectrum ratio, discrete wavelet transform and so on. Machine learning methods were used to classify these features, such as k-nearest neighbors, artificial neural network, gaussian mixture model, linear discriminant analysis, hidden Markov model, support vector machine and random forests. Although the promising performance of these feature-based methods have been shown, the complex process of feature extraction may result in the loss of useful information [21].

To solve the problem, researchers started to use deep learning-based approaches, such as convolutional neural network (CNN), to automatically learn features from a large amount of data. Ding [22] used a multiple-scale CNN structure to deduce information loss in feature extraction. Geng [23] proposed a deep CNN to classify instantaneous sEMG data images generated by a high-density electrodes matrix. Allard [6] transformed sEMG data to frequency spectrum images using Fast Fourier Transform, then used CNN to classify them. Wei [24] proposed a multi-channel CNN, and improved recognition accuracy by learning the correction between muscle and gesture. Shen [25] built a complex model that ensembled multiple CNN with a stacking method. Allard [26] combined CNN and transfer learning to decrease the data requirement of the training model for a new subject.

Recurrent neural network (RNN) is also a deep learning-based approach, which has been widely studied in the field of speech recognition and machine translation in recent years. Different from CNN which is good with hierarchical or spatial data and extracting unlabeled features, RNN is good at temporal or otherwise sequential data [27]. Simao [28] compared the performance between forward propagation neural network and RNN on the recognition of eight hand gestures. He [29] combined long short-term memory (LSTM) and multiple layer perceptron to learn features of static hand gesture. Nadia [30] used raw sEMG signal to recognize six gestures, which could adapt to new subjects. Furthermore, some researchers used the combination of CNN and RNN to recognize hand gesture. Wu [21] proposed a method to combine LSTM and CNN to recognize hand gesture. Xie [31] compared the performance of a single CNN model, single LSTM model and hybrid model to recognize different gestures. Hu [27] introduced an attention mechanism into a hybrid model of CNN and RNN to recognize gesture in five sEMG benchmark databases. 

However, to our knowledge, most of the existing RNN-based methods [27,28,29,30,31,32] still focus on hand gesture recognition, which recognize hand gestures based on the whole temporal sEMG data, but make a prediction by RNN, which has already been used in other fields [33,34,35,36] and has not been studied. In this paper, we propose a novel method by using RNN to predict hand gesture. The trained RNN model could output instantaneous prediction for the sEMG data from the previous sampling time step. The sEMG signals of 21 hand gestures of 13 subjects were recoded to train the model. In the test part, when the movement of a gesture starts, the model outputs an instantaneous prediction at every sampling time step. 

The main contributions of this paper are as follows:

- A hand gesture dataset containing 21 short-term gestures of 13 subjects is recorded by the Myo armband, which is publicly available on the Github that also includes our code (https://github.com/ChauncyHe/HandGesturePrediction).
- A novel RNN model to predict hand gesture is proposed, which is able to predict the gesture in the process of the gesture. When sEMG data points of 200 ms are used, which are generated after the motion start of the gesture, the accuracy could be about 89.6%.

The rest of this paper is organized as follows: Section 2 describes the sEMG sensors used in this work, the process of data acquisition, and the proposed method in detail. Section 3 presents the experimental results and analysis. Section 4 summarizes the paper.

## 2. Materials and Methods

### 2.1. sEMG Dataset

#### 2.1.1. Recording Device

**Figure 1:**
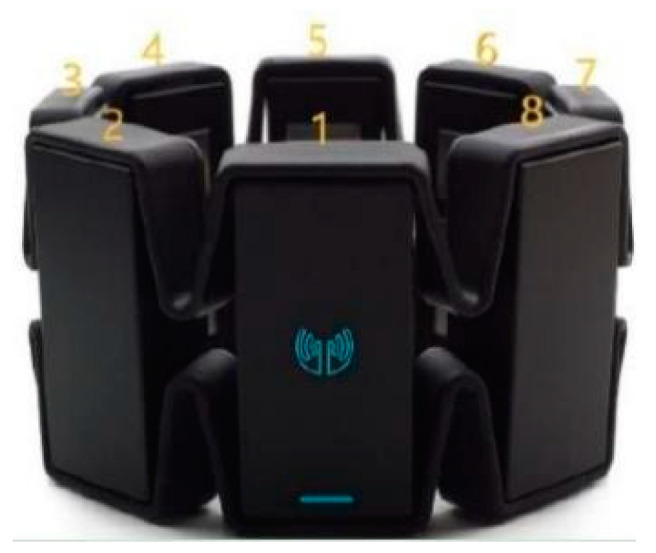
Myo armband.

There are various commercial devices or sensors to record sEMG signals, such as Thalmic Myo armband, Otto Bock 13e200 electrodes, Cometa Wave Plus system, Ag-AgCl Duo-Trode electrodes and Delsys Trigno system. Considering the portability in a real application scenario, we selected one low-cost wireless Myo armband from Thalmic Labs (Figure 1) to record sEMG data, which has been used to recognize hand gestures in many studies [37]. The Myo armband consists of a low-consumption ARM Cortex-M4 120 MHZ microprocessor, 8 dry electrodes and 9-axis inertial measurement unit (IMU). Its 8 non-intrusive dry electrodes can record sEMG signals generated by muscles at a sampling frequency of 200 Hz with 8 bits of resolution for each channel.

**Figure 2:**
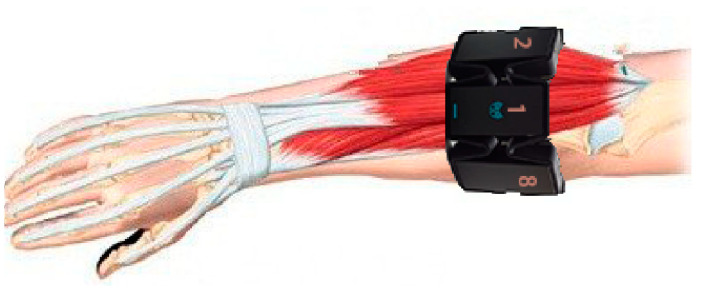
Wearing position and orientation.

As shown in Figure 2, the Myo armband wearing protocol is stipulated as follows: Firstly, the armband is located at a fixed position on the forearm of the right hand. The distance between the edge of the armband and the elbow joint is approximately the width of an index finger. Secondly, the wearing orientation is fixed. The first electrode is placed on the back of the forearm and aligned to the middle finger. Thirdly, the position and orientation of the armband is kept unchanged when recording data. Because the prediction model is subject-specified, the precisely same wearing position and orientation for all subjects is not necessary.

#### 2.1.2. Hand Gestures

**Figure 3:**
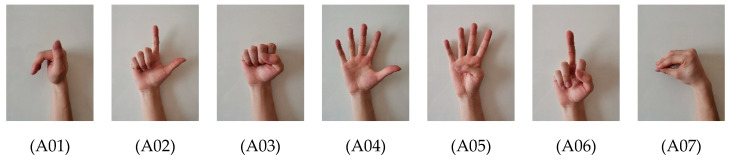

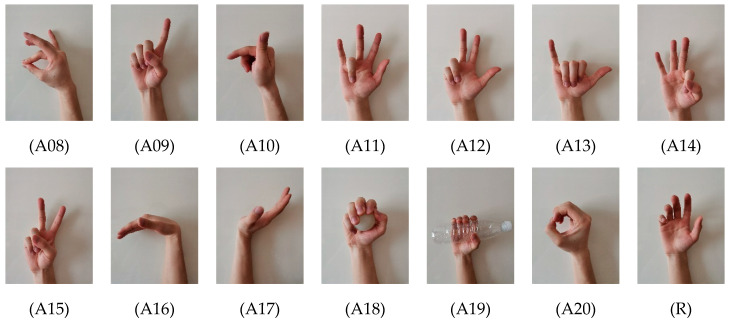
A01–A20 are 20 different hand gestures, and R represents the relaxation gesture. All these gestures (except R) are rapidly finished by going through a process of R-A-R within 2 s.

We designed 21 common gestures as shown in Figure 3. These gestures are mainly controlled by the traction of several major muscles on the forearm, and high visual similarity exists in some of these gestures. For example, grabbing a cylinder (A19) and grabbing a sphere (A18) are two similar behaviors. In addition, A09, A15, A14, A05 and A04 are five finger gestures corresponding to numbers 1–5, respectively. A20 is finished by bending five fingers into the shape of zero, which is also similar to a fist (A03). Most of these gestures are realized by the motion of the finger joints, while A16 and A17 are finished by the motion of the wrist joint. Relaxation gesture is regarded as the 21st gesture. Different from previous studies, all gestures are asked to be finished in the short term, which occurs within 2 s instead of maintaining more than 5 s.

#### 2.1.3. Acquisition Protocol

We recruited a total of 13 healthy right-handed volunteers (including 8 males and 5 females, aged from 23 to 25). Each subject wore the Myo armband on the right forearm near the elbow joint in accordance with the aforementioned wearing protocol. Before formal recording of data, the subject was instructed on how to perform each kind of hand gesture until the subject was able to perform all gestures by themselves without difficulty. Real-time sEMG data were transmitted from the Myo armband to a personal computer by a wireless receiver. The data of 30 repetitions of 21 kinds of hand gestures was recorded for each subject. Although the sampling time of each repetition lasted 2 s, a complete hand gesture was suggested to be finished in 0.5–1.5 s. Muscle fatigue will be caused by continuous multiple repetitions [38]. Therefore, to alleviate the harmful impact of muscle fatigue, a 2-s break between repetitions and a 5-min break between different gestures were reserved. In Table 1, a detailed data acquisition configuration for all subjects is shown. For each subject, a total of 630 samples were recorded. A sample is comprised of sEMG signal $X\in {\mathbb{R}}^{400\times 8}$ and corresponding label $Y\in {\mathbb{R}}^{400\times 21}$, where X is 8-channel sEMG data in 400-time steps and Y is the one-hot coding label of these time steps.

### 2.2. Methods

In this section, the details of the proposed method are described. It is worth noting that we do not perform any common data preprocessing on input sEMG signals such as filtering and rectification. That is to say, we will use raw sEMG signals to train the RNN model, which could give a category prediction result by using raw sEMG signals in the test stage. 

Firstly, before training the RNN model, motion detection which detects the start and end of a hand gesture is used to label the gesture. Then, the structure of the recurrent neural network model is described. Finally, post-processing is performed to obtain the final prediction result for a sample from instantaneous predictions generated at every time step. 

#### 2.2.1. Motion Detection

In the process of data recording, since subjects are only required to finish a complete hand gesture within 2 s, the gesture start time step $t_{s}$ and the end time step $t_{e}$ for each sample are both unknown. Motion detection is a key process to label gestures. There are many methods including moving average algorithm [3], standard deviation [5] and spectrogram technology [39]. In this study, we used the standard deviation of multi-channel sEMG signals in the time domain to detect the gesture. A sliding window was used to extract the average standard deviation of 8 channels.

(1) $$S_{1}\left[t,c\right]=\sqrt{\frac{1}{w}\sum_{t-w}^{t}{\left(X\left[t,c\right]-\frac{1}{w}\sum_{t-w}^{t}X\left[t,c\right]\right)}^{2}},$$

(2) $$S_{2}\left[t\right]=\frac{1}{N_{c}}\sum_{c=1}^{N_{c}}S_{1}\left[t,c\right].$$

where t=1,2,3,⋯,400 represents time steps, and c=1,2,3,⋯8 represents channels. w represents the width of the sliding window. X is the sEMG data of a sample, and $N_{C}$ represents the number of channels.

**Figure 4:**
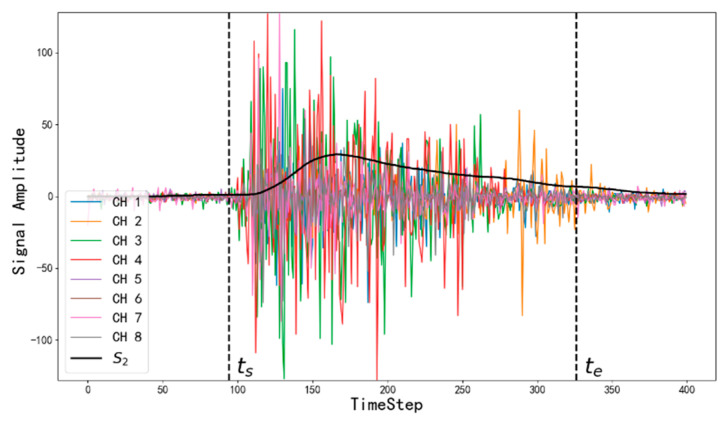
The curves of surface electromyographic (sEMG) signals and the motion detection result.

The sliding window is computing the standard deviation of the front w data points at time step t. To guarantee the consistency of array shape, zero-padding is used when t<w. Considering the sensitivity of each channel to gestures are different, an average operation on channels is performed to get a more robust detection (Figure 4). 

#### 2.2.2. Model Structure

**Figure 5:**
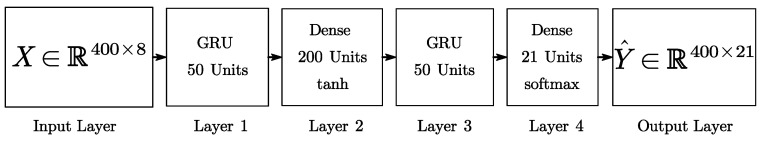
The model structure of recurrent neural network (RNN).

The RNN model is established by using gated recurrent units (GRU), a variant of recurrent neural units, as shown in Figure 5. The input layer is 8 channels raw sEMG data $X\in {\mathbb{R}}^{400\times 8}$ of a gesture sample. Layer 1 consists of 50 GRU units and uses the tanh activation function, which memorizes the signal change in the time domain. Layer 2 is a fully connected layer with 200 general units, and uses Tanh as the activation function. Layer 3 is also a recurrent layer that is the same as layer 1. Layer 4 has 21 units and uses SoftMax as the activation function to output probabilities of multiple categories. L1 or L2 regularization is not used in this study, and dropout is also not necessary by our experiments. For two recurrent layers, the sequence output is used, which guarantee the model output is also a sequence.

Because of the adding of GRU units in layer 1 and layer 3, the model output contains the time information as well. Model output $\hat{Y}\in {\mathbb{R}}^{400\times 21}$ is the changing process of the instantaneous prediction result with respect to the increasing time steps of sEMG data. In detail, $\hat{Y}\left[t,:\right]\in {\mathbb{R}}^{21}$ is an instantaneous prediction result at timestep t, which is represented by a probability vector obtained by layer 4. Additionally, according to the time characteristic of a unidirectional recurrent neural network, the instantaneous prediction result $\hat{Y}\left[t,:\right]$ is merely determined by sEMG data slice $X\left[1:t,:\right]\in {\mathbb{R}}^{t\times 21}$.

#### 2.2.3. Post-Processing

When the gesture start is detected at $t_{s}$ by motion detection, the model starts to output an instantaneous prediction result at every time step. In fact, when $t=t_{s}+M$, a total of M instantaneous prediction results are obtained. To improve the real-time performance of gesture prediction, a small number of m(m<M) instantaneous results are used to make a decision in post-processing. To get a final prediction category label from these m instantaneous prediction results, a simple and effective approach is to merely consider the last instantaneous result at $t=t_{s}+m$, which could be formed as the following:

(3) $$\hat{L}=\mathrm{argmax}\;Y\left[t_{s}:t_{s}+m,:\right].$$

where $t_{s}$ is the gesture start timestep of this sample, and m denotes how many time steps of sEMG that are used to predict. Then $\hat{L}\in \left\{1,2,\cdots ,21\right\}$ represents 21 different gestures. When m equals 40 timesteps, it means that the final prediction category label could be obtained with a delay of 200 ms after the hand starts to perform the gesture, instead of waiting for the end of the gesture. 

### 2.3. Training and Test Details

**Figure 6:**
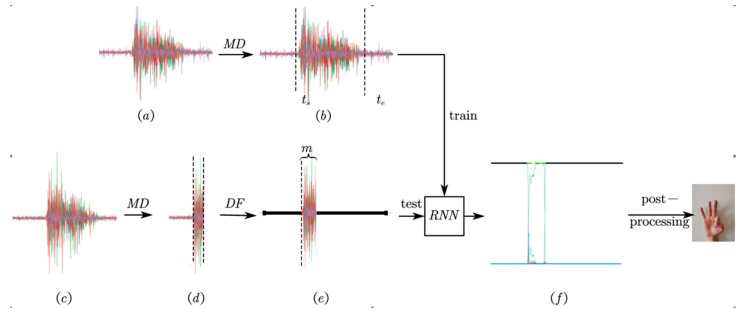
Flow diagram of the training stage. MD: motion detection. DF: data filling. (**a**) Training data. (**b**) Modify label of sample. (**c**) Test data. (**d**) Data after motion detection. (**e**) Prepare input data by DF. (**f**) The output of the RNN model.

Considering the relatively small number of samples for training a deep learning-based model with raw sEMG signals, stratified 5-folds cross-validation is used to partition datasets and evaluate model performance. Stratified partition guarantees enough sample numbers to train each category, and prevents resulting in an unbalanced model because of common random partitions. An average test accuracy on these 5 models is regarded as the generalization ability of the current model structure and hyper-parameters. Training details and test details are shown in Figure 6.

In the training stage, gesture start time step $t_{s}$ and gesture end time step $t_{e}$ of all training samples are detected to label the dataset by motion detection, and the RNN model is trained on the labelled dataset. We selected Adam optimizer to update model weights and used the cross entropy loss function to measure model error. The initial learning rate was set to 0.01 and a learning rate reducing mechanism was used. 

In the testing stage, when the gesture start is detected by motion detection, the model makes use of m time steps of record data after the gesture starts to predict hand gesture. As shown in Figure 6, record data obtained from motion detection are sent to the trained model after data filling which fills zero into the record data to make the input data $X\in {\mathbb{R}}^{400\times 8}$. Prediction results are obtained from the model output after post processing. 

After getting prediction category labels of every sample in the testing set by post-processing, we could obtain the evaluation performance of the model as usual classification tasks. Prediction accuracy of the model is determined by:

(4) $$ACC=\frac{\sum_{i=1}^{N_{S}}I\left({\hat{L}}_{i}=L_{i}\right)}{N_{S}}$$

where *N_s_* is the number of test samples, final category prediction result is represented by $\hat{L}\in \left\{0,1,\cdots ,20\right\}$, $L_{i}$ is the true category label of sample *i*, I(p) equals to 1 when p is true otherwise it equals to 0.

## 3. Results and Analysis

### 3.1. Accuracy Performance

**Figure 7:**
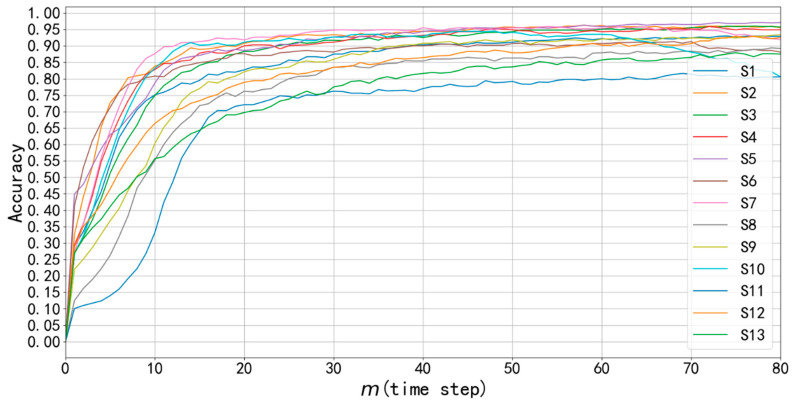
Accuracy with respect to m on 13 subjects. The horizontal axis m denotes how many time steps of sEMG data are used to predict gesture. Additionally, the vertical axis denotes the average accuracy of 5-fold cross validation.

Prediction accuracy of each subject is shown in Figure 7. The result shows that prediction accuracy increases with m, which conforms to the intuition that more known data points of gesture leads to a more reliable result. The average accuracy of all subjects on some major values of m are recorded in Table 2. When m=40 time steps ($T_{m}=200\;\mathrm{ms}$), the average prediction accuracy on testing set reached 89.6%. 

**Figure 8:**
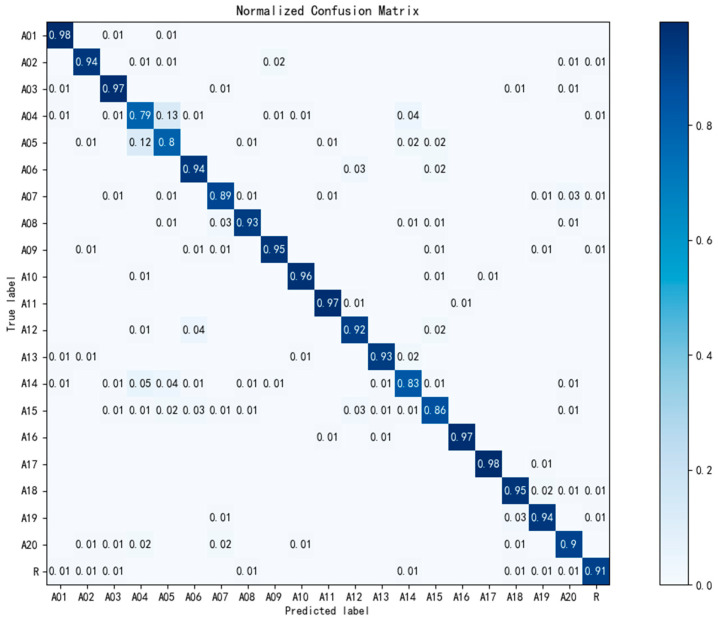
Hybrid test confusion matrix of all subjects.

To figure out the prediction performance of the model on each kind of hand gesture, the confusion matrix is plotted in Figure 8, on the condition that m=40 time steps. In order to more objectively express the model prediction performance on these 21 types of gestures, the confusion matrix is the hybrid of all subjects. From this figure, we found that several similar finger motion gestures representing numbers such as A04 (five), A05 (four), A14 (three) and A15 (two) were more likely to be confused with each other. On the contrary, A16 (wave in) and A17 (wave out) that only used the wrist joint, were relatively easy to predict. 

### 3.2. Real-Time Performance

**Figure 9:**
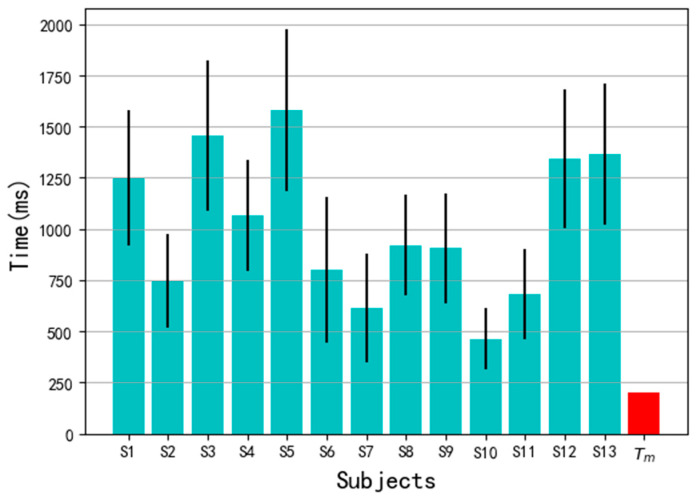
The motion time $T_{a}$ of each subject and $T_{m}=200\;\mathrm{ms}$.

For a hand gesture, the time $T_{a}=t_{e}-t_{s}$ represents the actual time span of a complete gesture. The average $T_{a}$ on all samples of a subject varies between 463 ms and 1582 ms as shown in Figure 9. When m=40 time steps, corresponding $T_{m}=200\;\mathrm{ms}$. From the figure, we find that $T_{m}\;\ll \;T_{a}$, which means the model will give prediction output after the gesture has started 200 ms before the gesture ends.

### 3.3. The Feasibility of Prediction

**Figure 10:**
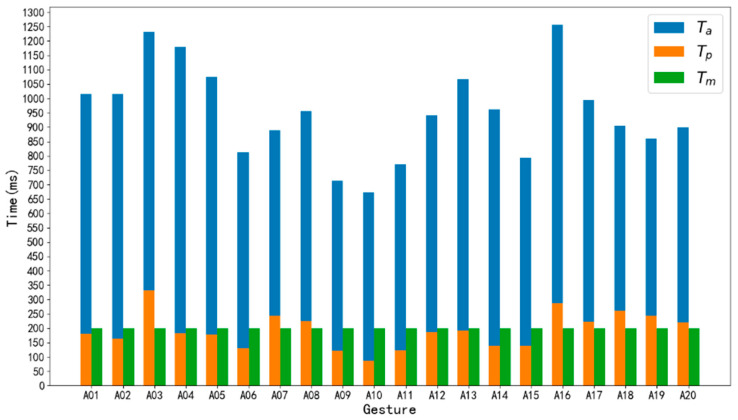
The comparison between $T_{a},T_{p}$ and $T_{m}$ of a subject.

According to [37], a gesture contains transient state and steady state, and transient state is generated when the gesture is in motion, while steady state is produced when the gesture is maintained. Classification of hand gestures in the transient state has lower accuracy than in the steady state. To explore whether the data in the transient state of a gesture is able to be used to predict gesture, *T_p_* is defined as the time of transient state and a comparison between $T_{a}$, $T_{p}$ and $T_{m}$ of a subject is illustrated in Figure 10. When $T_{m}=200\;\mathrm{ms}$, the accuracy of the model on the test set reached 89.6%. We can see for some drastic gestures such as A03 (fist) and A16 (wave in), transient time *T_p_* was longer than that of other gestures. Additionally, for these gestures, sEMG data only in *T_p_* were used to predict hand gestures. The results show that it is feasible to obtain a high prediction performance by using our RNN method.

### 3.4. Comparison with Other Methods

In this study, the proposed method is used to predict hand gestures and the real-time performance, which is more important. Table 3 shows the real-time performance and other conditions of the proposed model and other previous studies which used RNN methods. Most of these models were not real-time models, especially those methods which were a combination of LSTM and CNN. Nasri [30] made a very high validation accuracy on six gestures, but each gesture away repeated about 200 times for a subject, which is an extremely heavy workload. In addition, the use of a sliding window of 940 ms severely decreases the model’s real-time performance. He [21] used a sliding window of 400 ms but the accuracy of gestures was 75.5%. Compared to these studies, our method could obtain a real-time performance with a good accuracy. 

### 3.5. Limitations

We regret that, although the Myo armband is a popular low-cost device widely used in relevant studies in the past, it is unfortunately not commercially available since 2018 [39]. To our knowledge, there are some sEMG devices which use dry electrodes like the Myo armband, including Delsys Trigno system, gForce-100 Armband [40], etc. 

In addition, more concerns have to be taken into consideration in the application of real-time hand gesture prediction. In data acquisition, the wearing position and orientation of the Myo armband is fixed for each subject, so the trained subject-specified model is sensitive to the subject and wearing situation of the Myo armband in the test stage. Generally, the robustness of the model needs to be enhanced in further work.

## 4. Conclusions

This paper proposes a hand gesture prediction method based on raw multi-channel sEMG signals. Firstly, a dataset containing 30 repetitions of 21 short-term hand gestures of 13 subjects was collected with a Myo armband, in which each complete gesture was finished within 0.5–1.5 s. Then, a RNN model was built, which output an instantaneous probability distribution at every time step after the start of the gesture was detected. Finally, the prediction result was acquired by post-processing from these instantaneous outputs. Experimental results show that prediction accuracy by our model could reach about 89.6% using data of 200 ms, which can be collected after the start of the gesture. 

## Figures and Tables

**Table 1:** Acquisition configuration summary for the collection of datasets.

Acquisition Device	Myo Armband	Gestures	21
Sampling frequency	200 Hz	Repetition times	30
Channel number	8	Sampling time of a repetition	2 s
Subject number	13	Finish time of a gesture	0.5–1.5 s
Age range of subjects	22–26	Repetition interval	2 s
Health state	Intact subjects	Gesture interval	5 min

**Table 2:** Prediction accuracy with different m.

*m* (Time Steps)	20	40	60	80
$T_{m}$(*ms*)	100	200	300	400
Accuracy	83.8 ± 7.5%	89.6 ± 5.5%	91.5 ± 4.7%	90.8 ± 5.4%

**Table 3:** Comparison with state-of-the-art using RNN.

Work	Channels	Device	Sampling Rate (Hz)	Gestures	Repetition	Gesture Duration (s)	Subjects	Classifier	Accuracy (%)	RTP (ms)
Hu [27]	10	Myo(8+2)	100	52	10	5	27	LCNN	87.0	NRT
Xie [31]	16	Myo(8+8)	200	17	6	5	10	LCNN	83.6	NRT
Wu [21]	8	Myo(8)	200	5	12	5	NI	LCNN	98.0	NRT
Simao [28]	16	Myo(8+8)	200	8	NI	NI	NI	LSTM/GRU	95.0	NRT
Samadani [32]	12	Myo(8+4)	100	18	6	5	40	LSTM	89.5	NRT
Nasri [30]	8	Myo(8)	200	6	195	10	35	GRU	99.8	940
He [29]	12	Myo(8+4)	100	52	10	5	27	LSTM	75.5	400
Ours	8	Myo(8)	200	21	30	2	13	GRU	89.6	200

Note: RTP represents real-time performance, which usually can be denoted as the width of the sliding window. NI denotes the corresponding term is not indicated in the paper clearly. LCNN is the combination of LSTM and CNN. NRT means the method is not a real time approach.
